# Exposing Image Forgery by Detecting Consistency of Shadow

**DOI:** 10.1155/2014/364501

**Published:** 2014-03-13

**Authors:** Yongzhen Ke, Fan Qin, Weidong Min, Guiling Zhang

**Affiliations:** ^1^School of Computer Science and Software Engineering, Tianjin Polytechnic University, Tianjin 300387, China; ^2^Department of Logistics Management, Nankai University, Tianjin 300071, China

## Abstract

We propose two tampered image detection methods based on consistency of shadow. The first method is based on texture consistency of shadow for the first kind of splicing image, in which the shadow as well as main body is copied and pasted from another image. The suspicious region including shadow and nonshadow is first selected. Then texture features of the shadow region and the nonshadow region are extracted. Last, correlation function is used to measure the similarity of the two texture features. By comparing the similarity, we can judge whether the image is tampered. Due to the failure in detecting the second kind of splicing image, in which main body, its shadow, and surrounding regions are copied and pasted from another image, another method based on strength of light source of shadows is proposed. The two suspicious shadow regions are first selected. Then an efficient method is used to estimate the strength of light source of shadow. Last, the similarity of strength of light source of two shadows is measured by correlation function. By combining the two methods, we can detect forged image with shadows. Experimental results demonstrate that the proposed methods are effective despite using simplified model compared with the existing methods.

## 1. Introduction

With the advent of the Internet and low-price digital cameras, as well as powerful image editing software, ordinary users have more access to the tools of digital doctoring than ever before. This makes it more and more difficult for a viewer to check the authenticity of a given digital image. This urges us to find a way to distinguish the authentic and tampered photos. Digital image blind forensic technology is becoming a new hotspot in the field of multimedia security with a wide application prospect because of its advantages of identifying image authenticity and source without relying on any signature extraction or preembedded information.

Over the past few years, many approaches based on the consistency of image source have been developed to detect image forgeries. The approaches are based on the fact that natural images are usually obtained through data acquisition devices, which introduce uniform characteristics to the entire image, and, henceforth, the variation in the local characteristics across the image can be used to detect tampering. The characteristics include chromatic aberrations [1, 2], sensor pattern noise [3], color filter array interpolation [4, 5], consistency of camera response function [6, 7], and lighting inconsistencies [8, 9].

Shadows are the necessary part of an object when copying and pasting the object into a target image to maintain the integrity. When tampering with photos, they are integral parts of an image and following consistency of such properties in shadows can be used to detect image forgeries [10–12].

**Figure 1:**
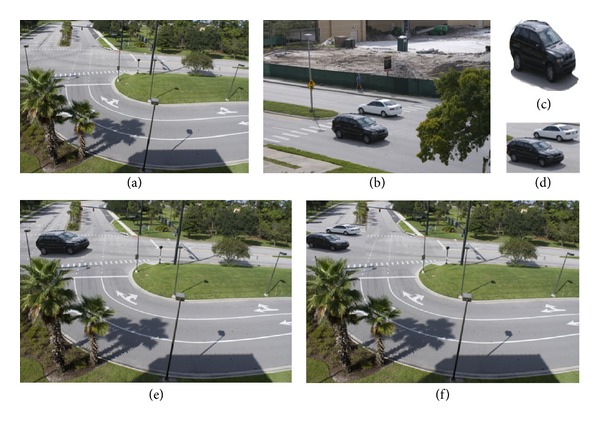
The samples of image forgery with shadows.

During the image forgeries, there are two cases about shadows. One is that the shadow as well as main body is copied and pasted from another image, as shown in Figure 1(e). Figures 1(a) and 1(b) are original images. The car and its shadow cropped from Figure 1(b) are shown in Figure 1(c). In another case as shown in Figure 1(f), the whole image block including main body, its shadow, and surrounding regions is copied and pasted from another image. Figure 1(d) shows the whole block cropped from Figure 1(b). Based on photometric properties of shadows in which the shadow does not obviously change the surface texture of object and the fact that both the position and strength of the light source can be estimated from shadows, this paper presents two tampered image detection methods through detecting consistency of shadows. First, the suspicious regions including shadow and nonshadow are selected by user. Then shadow region and nonshadow region are separated. Third, texture features and strength of light source of shadows are extracted. Last, correlation function is used to measure the similarity. By comparing the similarity, we can find whether there exists inconsistency and judge whether the image is tampered.

Compared with geometric constraint methods [10, 12], our methods just need a simple user interaction, while they need to select several key points in shadow using relatively complex user interface. Our method is also different from [11] in two aspects. First, our method focuses on two cases about shadow during the image forgeries. Second, our method can work in the situation in which a shadow is copied and pasted to another position in the same image.

This paper is organized as follows.  Section 2 presents the related works. The proposed methods are described in detail in Section 3. Experimental procedure and results are discussed in Section 4. Finally, Section 5 draws conclusions and discusses future work.

## 2. Related Work

Shadow detection and removal is an important preprocessing for improving performance of many computer vision algorithms, including segmentation, object detection, scene analysis, stereo, and tracking. Decomposition of a single image into a shadow image and a shadow-free image is a difficult problem. Most research is focused on modeling the differences in color, intensity, and texture of neighboring pixels or regions. In [13], they detected the shadow region based on the shadow density, which was defined as a measure of brightness. Then the shadow was removed by modifying the brightness and color. In the end, a smooth filter was used to correct boundaries between sunshine and shadow regions. Some of the most popular approaches in shadow removal were based on color constancy conditions as the lightness algorithms, in using a so-called illuminant invariant approach [14]. Instead of attempting to estimate the color of the scene illuminant, illuminant invariant methods attempted to simply remove its effect from an image. Some methods exploited the fact that regions under shadow retain most of their texture. Texture correlation was a potentially powerful method for detecting shadows as textures are highly distinctive, independent on colors, and robust to illumination changes [15].

Shadow as an important feature of digital images has already been used in image forgery detection [10–12]. Zhang et al. [10] introduced a method based on shadow geometry and shadow photometry for detecting photographic composites. Inconsistencies in the location of a cast shadow were used in [10], which placed several assumptions on the scene geometry; shadows were cast onto a planar ground plane and the objects casting shadows were vertical, relative to the ground plane. This method worked pretty well when the shadow receiving surface was flat and not textured. Photometric inconsistencies of illumination in shadows were used to detect inconsistent shadows in [11]. They formulated color characteristics of shadows measured by the shadow matte value and extracted the shadow boundaries and the penumbra shadow region in an image. Last, shadow matte values of shadows were estimated for each of the sampled shadows in an image and the consistency of them was used to inform whether the image was doctored. Kee et al. [12] described a geometric method to detect physically inconsistent arrangements of shadows in an image. This method combined multiple constraints from cast and attached shadows to constrain the projected location of a point light source in an image and can be used to determine whether there were physical consistencies with a single illuminating light source.

## 3. Materials and Methods

### 3.1. The Characteristics of Shadows

Shadows contain a wealth of information in digital images. There are many important visual cues of shadow for depth, shape, content, and lighting as described as follows [16–18].

- The value of shadow pixels must be low in all the RGB bands. Shadows are, in general, darker than their surrounding region.
- Shadows do not significantly change either the color or the surface texture of the background covered. Surface markings tend to continue across a shadow boundary under general viewing conditions.
- Shadow is always associated with the object that casts and the behavior of that object (e.g., if a person opens his arms, the shadow will reflect the motion and the shape of the person).
- Shadow shape is the projection of the object shape on the background. For an extended light source (not a point light source), the projection is unlikely to be perspective.
- Both the position and strength of the light source are known from shadows.
- Shadow size depends on the light source direction and the object height.

Our methods take the advantage of the properties that shadows do not obviously change the surface texture of object and the fact that the strength of the light source can be estimated from shadows.

### 3.2. Detection Method Based on Texture Consistency of Shadow

Because shadows do not significantly change the surface texture of the background covered as mentioned in Section 3.1, inconsistency of the surface texture between shadows region and nonshadows region implies a forgery in a splicing image, in which the shadow as well as main body is copied and pasted from another image during forgery. In other words, the shadow regions should have the same or similar texture with their adjacent nonshadow regions in authorized image.

**Figure 2:**
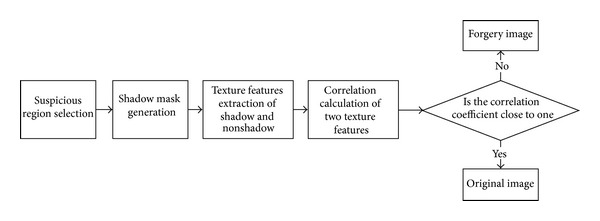
Image forgery detection method based on texture consistency of shadow.

Image forgery detection method based on texture consistency of shadow is shown in Figure 2. First, the suspicious region including shadow and nonshadow is selected by user through user interface. Then, the shadow mask is used to separate the shadow regions and nonshadow regions. Third, the texture's features of two regions laid inside and outside the shadow are extracted, respectively. Last, calculation of similarity between the texture features is applied to decide whether the input image is an original image or a forgery image.

In order to obtain a shadow mask, the gray thresh function based on Otsu's method [19], which chooses the threshold to minimize the intraclass variance of the black and white pixels, is used to convert an intensity image into a binary image.

**Figure 3:**
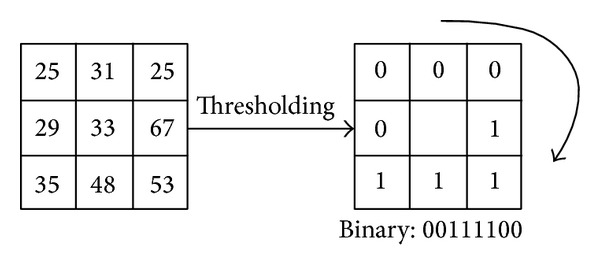
Basic LBP operator.

Many texture feature extraction methods, such as Gray-Level Cooccurrence Matrix, Local Binary Pattern, Gabor Filtering, and Difference Matrix, have been developed in the past several decades. Due to its texture discriminative property and its very low computational cost, LBP (Local Binary Pattern) is becoming very popular in pattern recognition. So, LBP is used in this paper. LBP was introduced by Ojala et al. in 1996 [20] for texture classification. Basic LBP operator is a computational efficient operator. Taking each pixel as a threshold, the operator transferred its 3 × 3 neighborhood into an 8-bit binary code, as shown in Figure 3.

The decimal form of the resulting 8-bit word (LBP code) can be expressed as follows:

(1) $$\begin{array}{c} \text{LBP}=\sum_{i=0}^{7}B\left(p_{i}-p_{c}\right)2^{i}, \end{array}$$

where *p*_*i*_ corresponds to the grey value of the center pixel (*xc*, *yc*), *p*_c_ corresponds to the grey values of the 8 surrounding pixels, and function *B*(*x*) is defined as

(2) $$\begin{array}{c} B\left(x\right)=\{\begin{array}{cc} 1, & \\ & \frac{x\geq 0,}{x<0.}\\ 0, & \end{array} \end{array}$$

In literature [21], Heikkilä et al. introduced the CS-LBP operator for region description which is more efficient than LBP.

The scheme functions of LBP and CS-LBP are given as follows:

(3) $$\begin{array}{c} S_{\text{LBP}}\left(p_{i},p_{c}\right)=\{\begin{array}{cc} 1, & p_{i}>p_{c},\\ 0, & \text{otherwise}, \end{array}\\ S_{\text{CS-LBP}}\left(p_{i},p_{i+(p/2)}\right)=\{\begin{array}{cc} 1, & p_{i}-p_{i+(p/2)}>T,\\ 0, & \text{otherwise}, \end{array} \end{array}$$

where *p*_*i*_, *p*_*i*+(*p*/2)_ and *p*_*c*_ correspond to the gray-level of center-symmetric pairs of pixels and the center pixel on a circle of radius *R* and *T* is the threshold for the CS-LBP descriptor. The binary patterns of LBP and CS-LBP are calculated as

(4) $$\begin{array}{c} \text{LB}{\text{P}}_{P,R}\left(x,y\right)=\sum_{i=0}^{P-1}S_{\text{LBP}}\left(p_{i},p_{c}\right)\times 2^{i},\\ {\text{CS-LBP}}_{P,R,T}\left(x,y\right)=\sum_{i=0}^{(P/2)-1}S_{\text{CS-LBP}}\left(p_{i},p_{i+(p/2)}\right)\times 2^{i}, \end{array}$$

where (*x*, *y*) denotes the coordinates of a pixel.

After texture features of shadow and nonshadow region are extracted, a simple method is used to measure the similarity of texture features. Let *X* be the texture feature of shadow region and *Y* be the texture feature of nonshadow region, where *X* and *Y* are vectors of the same size *n*. The two-dimensional correlation coefficient *r* between *X* and *Y* is defined as follows:

(5) $$\begin{array}{c} r=\frac{\sum_{n}\left(X_{i}-\bar{X}\right)\left(Y_{i}-\bar{Y}\right)}{\sqrt{\left(\sum_{n}{\left(X_{i}-\bar{X}\right)}^{2}\right)\left(\sum_{n}{\left(Y_{i}-\bar{Y}\right)}^{2}\right)}}, \end{array}$$

where $\bar{X}=\text{means}(X)$, and $\bar{Y}=\text{means}(Y)$.

If the correlation coefficient *r* is not close to one, we can find that there exists an inconsistency and suspect that the shadow region is most likely a tampered region. Generally, the area of the tampered region is usually smaller than their authentic counterparts. To improve accuracy, several shadow regions are selected in a suspicious image for measurements of texture similarity. The shadow region with *r*, which is different from others, is treated as tampered region.

### 3.3. Detection Method Based on Strength Consistency of Light Source of Shadows

**Figure 4:**
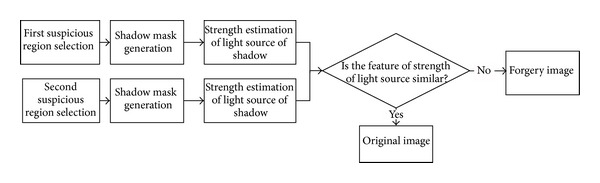
Image forgery detection method based on strength consistency of light source of shadows.

Natural images usually introduce uniform characteristics to the entire image. The strength of the light source obtained from shadows should be consistent in a natural image. During the image forgeries, the whole image block including main body, its shadow, and surrounding regions is often copied and pasted from another image. Henceforth, the variation of strength of the light source obtained from shadows can be used to detect tampering. By comparing the strength of the light source of two shadows, we can find whether there exists an inconsistency and suspect whether the image is tampered. Image forgery detection method based on strength consistency of light source of shadows, similar to [10, 11], is shown in Figure 4. The two suspicious regions including shadow area and nonshadow area are first selected by user through user interface. Then, a simple and efficient method is used to estimate the strength of light source of shadow. Last, the similarity of strength of light source of two shadows is measured by correlation function.

In this paper, we adopt a simple shadow model, where there are two types of light sources: direct light and environment light [22]. Direct light comes directly from the source (e.g., the sun), while environment light is from reflections of surrounding surfaces. Nonshadow areas are lit by both direct light and environment light, while for shadow areas, part or all of the direct light is occluded. The shadow model can be represented by following formula:

(6) $$\begin{array}{c} I_{i}=\left(t_{i}\cos\theta_{i}L_{d}+L_{e}\right)R_{i}, \end{array}$$

where *I*_*i*_ represents the value for the *i*th pixel in RGB space, similarly, both *L*_*d*_ and *L*_*e*_ represent the intensity of the direct light and environment light, also measured in RGB space, *R*_*i*_ is the surface reflectance of that pixel, *θ*_*i*_ is the angle between the direct lighting direction and the surface norm, and *t*_*i*_ is the attenuation factor of the direct light with a value between [0, 1]. When *t*_*i*_ = 1, the pixel is in a sunshine region, and when *t*_*i*_ = 0, the pixel is in an umbra; otherwise, the area is in a penumbra (0 < *t*_*i*_ < 1). For a shadow-free image, every pixel is lit by both direct light and environment light and can be expressed as

(7) $$\begin{array}{c} I_{i}^{\text{shadow}\_\text{free}}=\left(L_{d}\cos\theta_{i}+L_{e}\right)R_{i}. \end{array}$$

We define *k*_*i*_ = *t*_*i*_cos⁡*θ*_*i*_ as the shadow coefficient for the *i*th pixel and *r* = *L*_*d*_/*L*_*e*_ as the ratio between direct light and environment light. If *k*_*i*_ = 1 means that the object point is in nonshadow regions, an image *I* with shadow can be seen as the linear combination of a shadow-free image *L*_*d*_ *R* + *L*_*e*_ *R* and a shadow image *L*_*e*_ *R*, by rewriting the shadow formulation given in (6) as

(8) $$\begin{array}{c} I_{i}=k_{i}\left(L_{d}R_{i}+L_{e}R_{i}\right)+\left(1-k_{i}\right)L_{e}R_{i}, \end{array}$$

where *I*_*i*_ is the RGB value of the *i*th pixel of the original image *I*.

Based on this shadow model, the new pixel value of shadow-free image is given by

(9) Iishadow_free=(Ld+Le)Ri=(Ld+Le)RikiLd+LekiLd+Le=(kiLd+Le)RiLd+LekiLd+Le=r+1kir+1Ii.

In order to calculate the ratio *r* between direct light and environment light, we check for adjacent shadow/nonshadow pairs along the shadow boundary. These patches are of the same material and reflectance. Based on the lighting model (formula (6)), for two pixels with the same reflectance, we have

(10) $$\begin{array}{c} I_{i}=\left(k_{i}L_{d}+L_{e}\right)R_{i},\\ I_{j}=\left(k_{j}L_{d}+L_{e}\right)R_{j}. \end{array}$$

With *R*_*i*_ = *R*_*j*_, where *I*_*i*_ is shadow region and *I*_*j*_ is nonshadow region, from the above equations, we can arrive at

(11) $$\begin{array}{c} r=\frac{L_{d}}{L_{e}}=\frac{I_{j}-I_{i}}{I_{j}k_{j}-I_{i}k_{i}}. \end{array}$$

We consider a special case that *k*_*i*_ is zero for umbra region *I*_*i*_ and *k*_*j*_ is one for nonshadow region *I*_*j*_. Based on formulas (9) and (11), we can estimate the strength of light source of shadow as follows:

(12) Iishadow=Ii−Iishadow_free=Ii−r+1kir+1Ii=ki−rkir+1Ii≅−rIi≅Ii−IjIjIi.

Four features of strength of light source of umbra region *I*_*i*_^shadow^ including mean, std, skewness, and kurtosis are extracted to measure the similarity of two shadows. Similar to Section 3.2, if the correlation coefficient *r* is not close to one, we can suspect that the image is most likely tampered.

## 4. Results and Discussion

In this section, we utilize the proposed methods to image forgery detection and verify the effectiveness of our proposed methods using real photos. Some experiment images are selected from the shadow detection dataset in [22, 23], and others are collected by authors. All experimental images are manipulated using Photoshop and saved in JPEG format. We made simple user interface using Matlab software.

### 4.1. Detection Results Based on Texture Consistency of Shadow

**Figure 5:**
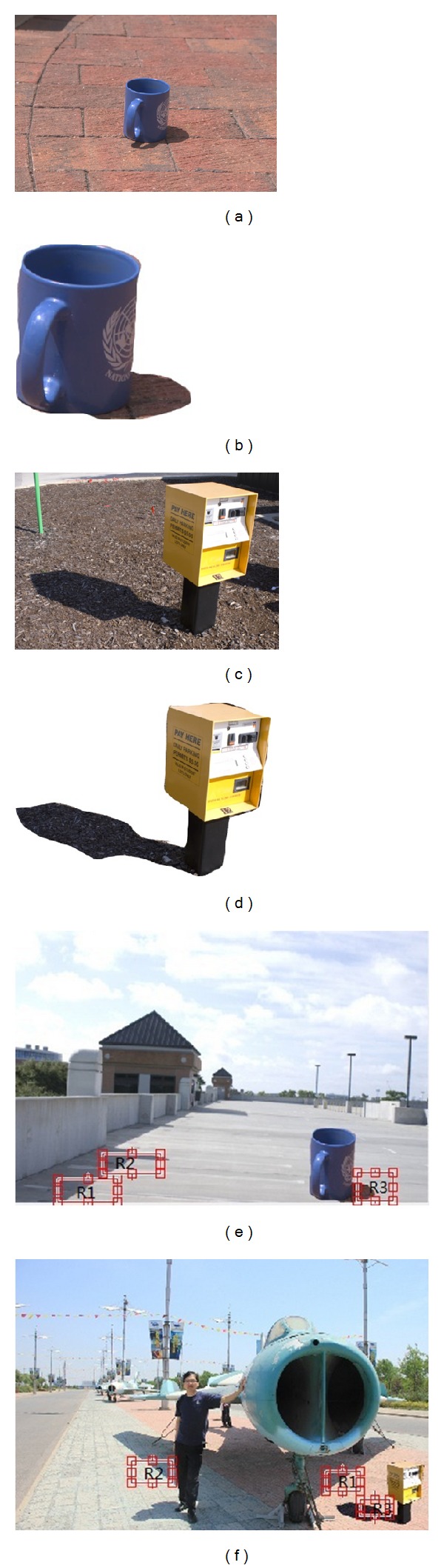
Examples of forged image where shadow as well as main body is copied and pasted from another image.

**Figure 6:**
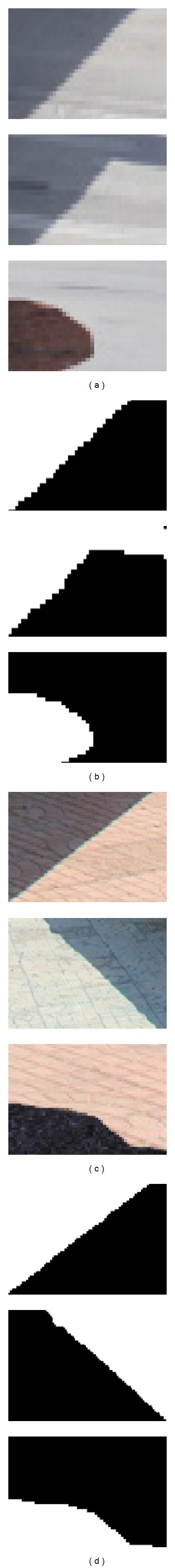
Sampled shadow regions and shadow mask.

We present image forgery detection results to show the efficacy of the proposed methods. Figures 5(a) and 5(c) are original images. Figures 5(b) and 5(d) are shadow regions cropped from Figures 5(a) and 5(c), respectively. Figures 5(e) and 5(f) show examples of forged images where shadow as well as main body is copied and pasted from another image. Three shadows in each image are sampled and marked by red boxes, respectively. In Figure 6, column (a) and column (c) gives the shadow region sampled from Figures 5(e) and 5(f), respectively. The top row (R1) and second row (R2) are authorized regions from original image and the last row (R3) is a suspicious region from another image. Columns (b) and (d) show the detected shadow mask.

Table 1 shows the results of similarity between texture features of two regions laid inside and outside shadow in Figure 6. The true regions (R1 and R2) have high similarity value with higher than 0.99, but the fake region (R3) has low value with lower than 0.95. Experiments on more images have been done, producing similar results. Due to the limit of the paper length, we do not show more results. From our experiments, it is observed that the proposed method can correctly identify tampered image region when the texture of cropped shadow region is different with the texture of background image. We also find that it is more difficult to correctly locate tampered region with increasing texture similarity between shadow region and nonshadow region. But another detection method based on strength consistency of light source of shadows can be used to improve performance. The result is illustrated in detail in Section 4.3.

**Figure 7:**
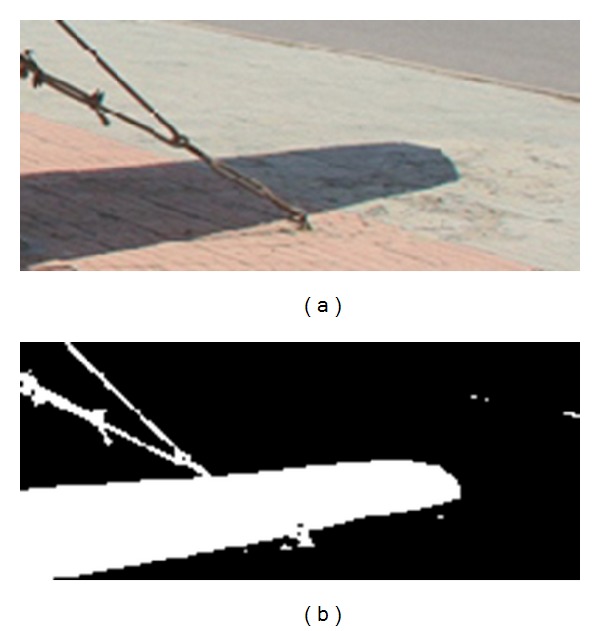
A failure case for our method.

Figure 7 is an image region sampled from the original image that demonstrates a failure case for our method. The similarity value *r* in Figure 7 is 0.92415. Because the shadow region cropped original image includes two kinds of textures, the similarity between texture features of two regions laid inside and outside shadow is low. Therefore, our method relies on a user's correctly selecting shadow region. A key step in applying our method is for the analyst to select a set of shadows from the image which only includes one kind of texture. A poor selection of shadow could, of course, lead to a failure in detecting a manipulated image.

### 4.2. Detection Results Based on Strength Consistency of Light Source of Shadows

**Figure 8:**
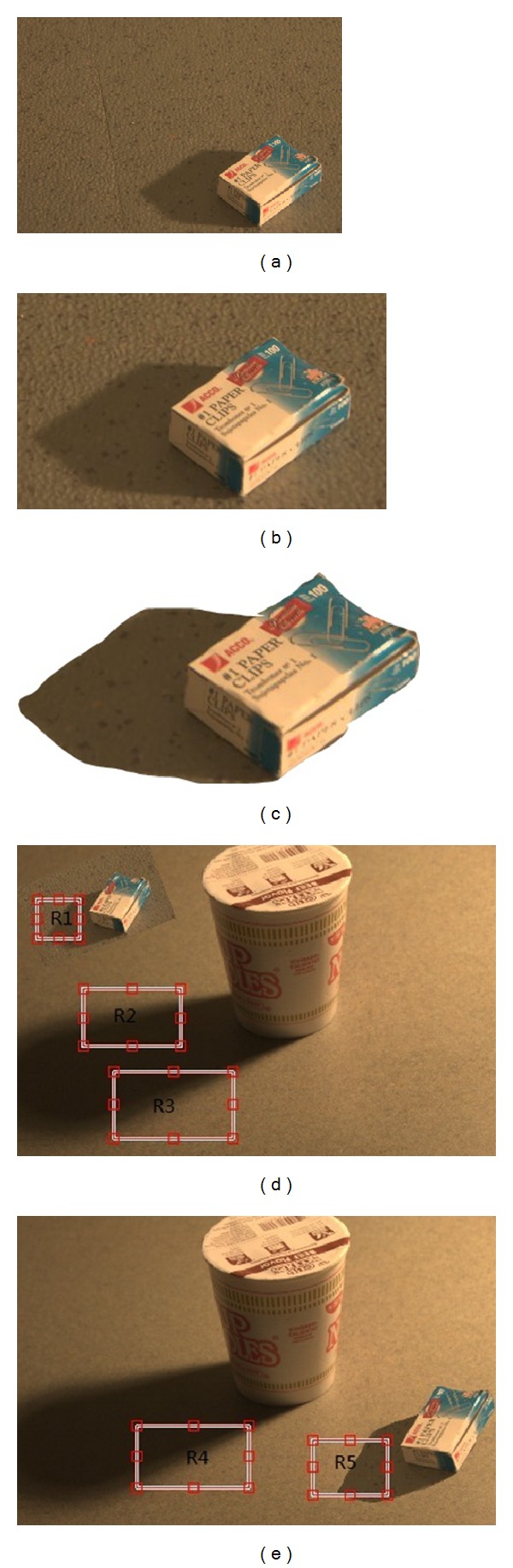
Examples of forged image with shadow.

**Figure 9:**
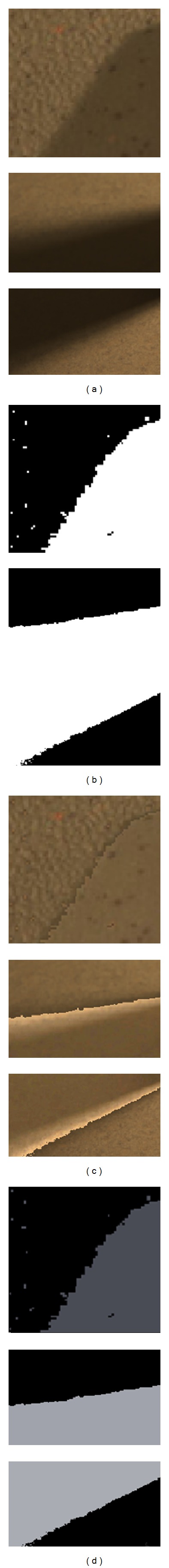
Sampled shadow regions, detected shadow mask, shadow-free image, and strength of shadow.

Figure 8(a) is an original image. Local image block and shadow region cropped from Figure 8(a) are shown in Figures 8(b) and 8(c).  Figure 8(d) shows an example of composite images where the whole image block including main body, its shadow, and surrounding regions is copied and pasted from another image. Three shadows in Figure 8(d) are sampled and marked by red boxes, respectively. In Figure 9, column (a) is sampled shadow region, column (b) is detected shadow mask, column (c) is shadow-free image, and column (d) is strength of shadow. The top row (R1) is a suspicious region from another image, and the second row (R2) and last row (R3) are authorized regions from original image.

Table 2 shows the results of similarity between two shadow regions in Figure 9. From Table 2, we can find that the similarity of strength of light source between the suspicious shadow region (R1) and authorized regions (R2 and R3) are 0.95972 and 0.94771, respectively, while the similarity of strength of light source between two authorized regions (R2 and R3) is 0.9999. More experiment results show that the proposed method can correctly identify tampered image.

### 4.3. Detection Results through Combining Two Methods

We also finished experiments combining two methods to improve detection performance.  Figure 8(e) is a tampered image, where the box and its shadow are copied and pasted from Figure 8(a). Two shadow regions (R4 and R5) are sampled. Using detection method based texture consistency of shadow, similarity between texture features of two regions laid inside and outsides of the shadow in R4 and R5 are 0.99648 and 0.99537, respectively. Because two shadow regions (R4 and R5) have very similar textures, it is difficult to identify tampered image region. Thereafter, based on our second method, it is easy to judge that Figure 8(e) is a tampered image because the similarity of strength of light source between R4 and R5 is computed as 0.76554.

**Figure 10:**
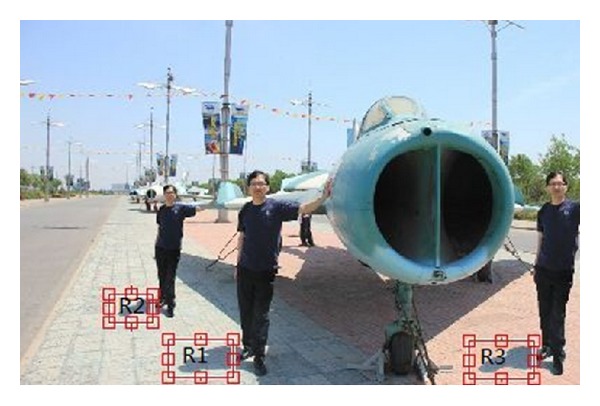
Examples of forged image with shadow.

Our second method and Liu's method [11] would fail in detecting the tampered image, where the man and its shadow (R1) are copied and pasted to another position (R2 and R3) in the same image as shown in Figure 10.  Table 3 shows the results of similarity between texture features of shadow and nonshadow regions, and similarity of strength of light source between two shadow regions in Figure 10. The similarity of strength of light source between R1, R2, and R3 is computed as 0.99981, 0.99033, and 0.9919, respectively. However, based on our first method, it is easy to locate the tampered region because the similarity between texture features of two regions laid inside and outside of the shadow in authorized regions (R1) is 0.99769, but the similarity between texture features in forged regions (R2 and R3) is 0.91228 and 0.94606, respectively.

The results above show that combining the proposed two methods can correctly locate tampered image region.

## 5. Conclusions

Based on consistency of shadow, two forgery image detection methods are proposed in this paper. The first method is based on texture consistency of shadow for splicing image, in which the shadow as well as main body is copied and pasted from another image during forgery. Another method is based on strength of light source of shadow for splicing image, in which main body, its shadow, and surrounding regions are copied and pasted from another image during forgery. Experimental results show that the proposed methods are effective despite using simplified model compared with existing methods. Though our method can identify whether an image is tampered, one limitation of our method is that it can only detect the tampered image with shadows. As pointed out by many other authors, there is no single technique to detect all kinds of image forgery. In the future, we will continue to optimize the methods and integrate our methods with other methods for more stable detection.

## Figures and Tables

**Table 1:** Detection result on image in Figure 6.

Rows in Figure 6	Similarity (*r*) between texture features of two regions laid inside and outside the shadow in Figure 6(a)	Similarity (*r*) between texture feature of two regions laid inside and outside the shadow in Figure 6(c)	True or fake
The top row (R1)	0.99452	0.99659	T
The second row (R2)	0.99554	0.99249	T
The last row (R3)	0.93334	0.94428	F

**Table 2:** Detection results on image in Figure 9.

Sampled region one	Sampled region two	Similarity of strength of light source between sampled shadow regions one and two
R1	R2	0.95972
R1	R3	0.94771
R2	R3	0.9999

**Table 3:** Detection results on image in Figure 10.

Sampled region one (similarity between texture features)	Sampled region two (similarity between texture features)	Similarity of strength of light source between sampled shadow regions one and two
R1 (0.99769)	R2 (0.91228)	0.99981
R1 (0.99769)	R3 (0.94606)	0.99033
R2 (0.91228)	R3 (0.94606)	0.9919
